# Association between Mediterranean dietary pattern with sleep duration, sleep quality and brain derived neurotrophic factor (BDNF) in Iranian adults

**DOI:** 10.1038/s41598-023-40625-4

**Published:** 2023-08-18

**Authors:** Sobhan Mohammadi, Keyhan Lotfi, Elahe Mokhtari, Zahra Hajhashemy, Zahra Heidari, Parvane Saneei

**Affiliations:** 1 Department of Community Nutrition, School of Nutrition and Food Science, Nutrition and Food Security Research Center, Isfahan University of Medical Sciences, PO Box 81745-151, Isfahan, Iran; 2 Department of Community Nutrition, School of Nutritional Sciences and Dietetics, Tehran University of Medical Sciences, Tehran, Iran; 3 Department of Biostatistics and Epidemiology, School of Health, Isfahan University of Medical Sciences, Isfahan, Iran

**Keywords:** Nutrition, Neurological disorders, Circadian rhythms and sleep

## Abstract

Data on the association between Mediterranean diet, sleep and brain-derived neurotrophic factor (BDNF) were limited in Middle Eastern populations. We examined the association between Mediterranean dietary pattern with sleep quality/quantity, and serum BDNF in Iranian adults. This cross-sectional study was performed among 535 middle-aged adults (54% men), selected by multistage cluster random sampling method. The Pittsburgh sleep quality index and a validated food frequency questionnaire were used to assess sleep quality, sleep quantity, and Mediterranean diet score (MDS). Twelve-hour fasting blood samples were taken to evaluate serum BDNF values. Participants in the highest tertile of MDS, in comparison to those in the lowest tertile, had lower odds of having short sleep (OR = 0.44, 95%CI: 0.21–0.91) and poor sleep quality (OR = 0.48, 95%CI: 0.22–0.96), after adjustment for potential confounders. Among specific domains of sleep quality, lower odds of subjective sleep quality, sleep latency, and daytime dysfunction were associated with increased MDS. Higher adherence to MDS among individuals with overweight or obesity reduced the odds of having short sleep; this relation was not seen among individuals with normal weight. In contrast, the association between sleep quality and the MDS was significant in individuals with normal weight, but not those with overweight or obesity. Participants with higher adherence to MDS had lower odds for low BDNF values; however, this relation was not statistically significant. Overall, Iranian adults with a higher adherence to MDS had considerably lower odds of having short sleep and poor sleep quality. BDNF would not be an intermediate molecule for this connection.

## Introduction

Short sleep duration or poor sleep quality are among the most common health problems that can reduce efficiency and quality of life^1^. Convincing evidence has been suggested that poor sleep quality and quantity could be negatively related to chronic conditions such as type 2 diabetes^2^, metabolic syndrome^3^, frailty^4^, cardiovascular disease^5^, and obesity^5^. Almost one-third of the general world population -including the Iranian society- suffers from sleep problems^6,7^, which increase with age and occur more in women than men^7,8^.

Lifestyle modifications and various environmental factors such as diet, physical activity, and weight status affect quality and/or quantity of sleep^1,9^. Previous investigations have shown associations between healthy diets and both sleep quality and quantity^10–12^. A variety of foods and food components have been identified to improve sleep quality and duration^13,14^. Tryptophan and foods rich in this amino acid, as well as dietary sources of melatonin, are regarded to be food items that improve sleep habits and are associated with a better sleep quality^15,16^. There is also a growing interest on the Mediterranean diet (MD) pattern for promoting sleep health^17,18^. Mediterranean diet supports intake of nutrient-dense fruits and vegetables, as well as foods high in plant-based protein and unsaturated fats, which can commonly improve sleep health^11,19^.

The favorable effects of the MD pattern may be attributed to the high amount of antioxidants sources and healthy fats, which improve insulin sensitivity and reduce inflammatory responses; so, this diet has been documented as a healthy diet^20,21^, but findings of its association with sleep are contradictory^17,18,22^. In a cohort study, more adherence to the MD pattern among Spanish elders was associated with lower risk of ≥ 2h/night decreased or increased in sleep duration, and poor sleep quality^17^. However, a longitudinal study showed that the MD is associated with a better sleep quality in the elderly adults, but no significant association was observed in case of sleep duration^22^. A cross-sectional study revealed a positive association between sleep quality and the MD among individuals with normal weight or overweight, whereas no relation was found in participants with obesity^11^.

Brain-derived neurotrophic factor (BDNF) is a component of neurotrophic factor family that improves survival and growth of brain neurons^23^. Studies have not reported a specific cut-point for an optimal BDNF level. Several human and animal studies suggested an association between BDNF and sleep quality^24^. Some previous studies discovered a direct association of BDNF with synaptic flexibility induced by wakefulness activities and hemostatic sleep response^25,26^. Another study on female nurses found that serum BDNF levels were substantially lower in individuals who had short sleep duration^27^. Also, an investigation among female rats revealed that sleep deprivation decreased concentrations of BDNF, significantly^28^.

Serum levels of BDNF are affected by environmental factors, particularly diet. Several dietary factors such as fatty acids, vitamin E and polyphenols have been assessed in relation to BDNF levels through animal and human studies^29–31^. In a clinical trial, the effect of the MD plus nuts or olive oil on plasma BDNF levels was evaluated among individuals with depression; compared to the low-fat diet as the control group, the group assigned to the MD plus nuts had a lower odds of having low BDNF values^32^. Nevertheless, there is no further evidence examining this relation.

Most previous studies investigating dietary patterns in relation to quality and quantity of sleep have been performed in European and American societies. Also, studies have not assessed differences among individuals with normal weight and those with overweight/obesity, as an important underlying factor for sleep quality and duration^33^. The relationship between the MD with BDNF as well as quality and quantity of sleep has not been studied among Iranian adults. Investigating these relations among Iranian population is worthwhile as dietary intakes of them are much different from populations of Western countries. Also, it seems that BDNF might be an intermediate factor in the association between MD and sleep quality and duration. Therefore, this study aimed to investigate the relationship between the MD and serum BDNF levels and quality and quantity of sleep in Iranian adults. We hypothesized that higher adherence to the MD could be related to a better quality and quantity of sleep as well as BDNF status.

## Methods and materials

### Study design and participants

This cross-sectional study was conducted on a representative sample of individuals in a large city in the center of Iran, in the year of 2021. Taking 35% prevalence of sleep deprivation among Iranian adults^34^, a power of 80%, type I error of 5%, and precision (d) of 4.1% into account, we required at least 519 participants for this study. Data were not collected during COVID-19 waves or quarantine time. However, given the possibility of a low response rate due to the prevalence of the COVID-19 pandemic during data collection, 600 individuals were invited to participate in the study. From 20 schools selected by the use of a multistage cluster random-sampling procedure from the city of Isfahan, 600 adults (from both genders) with an age range of 20–65 years were invited to the study. All adults working in the selected schools, including staff, teachers, school managers, assistants, and crews were included to obtain a representative sample of the general adult population with different socioeconomic levels. However, individuals with the following features were not eligible for the study: (1) being pregnant or breastfeeding; (2) having a history of cancer, type 1 diabetes, heart disease, or stroke; (3) following a weight loss (calorie-restricted) diet. Among invited participants, 543 agreed to take part in our study (response rate: 90.5%). We ruled out individuals with the following criteria: (1) left more than 70 items blank on the food frequency questionnaire (n = 4); (2) reporting energy intake outside of the range of 800–4200 kcal/day (as under-reporters and over-reporters^35^) (n = 3); (3) refusing to take a blood sample (n = 1). Therefore, 535 people were involved in the current analysis. All research was performed in accordance with relevant guidelines/regulations (the 1975 Declaration of Helsinki). Each participant signed an informed consent form. The Isfahan University of Medical Sciences Ethics Committee has ethically approved the protocol of the study (no. IR.MUI.RESEARCH.REC.1399.613).

### Assessment of dietary intake

We evaluated usual dietary intake of participants via a validated Willett-format semi-quantitative 168-item food frequency questionnaire (FFQ)^36^. Reasonable associations between dietary intakes evaluated by the applied FFQ and those obtained from several 24-h dietary recalls were found in a prior validation study on 132 middle-aged Iranian adults^36^. Dietary intakes determined by the FFQ and twelve 24-h food recalls were reasonably correlated for total calories (r = 0.55), proteins (r = 0.65), fat (r = 0.59), fiber (r = 0.67), and magnesium (r = 0.65). The reliability of the FFQ was evaluated by comparing the nutritional intakes acquired from the FFQ on two times 1-year apart. Overall, the information showed that this FFQ may provide reasonably accurate and reliable measurements of usual dietary intakes of Iranian adults^36^. The study participants were instructed by a trained dietitian to fill out the FFQ by indicating how frequently and how much they consumed each food item in the last year. After that, using standard home measurements, the portion sizes of consumed foods were converted to grams per day^37^. Then, we entered all food items into Nutritionist IV software to calculate daily consumption of all nutrients and energy intake.

### Assessment of adherence to Mediterranean diet

Following the methodology developed by Trichopoulou et al.^38^, we determined the Mediterranean dietary score (MDS) based on nine components [including fruits, vegetables, nuts, legumes, fish, monounsaturated fatty acids (MUFAs) to saturated fatty acids (SFAs) ratio, grains, meats, and dairy products]. First, we obtained energy-adjusted intakes of these items by use of residual method^35^. In this method, food groups were regressed on total energy intake. The residuals obtained from this regression model were summed up with the mean food group intakes to have energy-adjusted estimates. Then, for each food item, participants received a score of one if their intake of vegetables, fruits, legumes, nuts, fish, and MUFAs/SFAs ratio was higher than the median, and consumption of grains, meats, and dairy products was lower than the median. Participants who had more median intake of grains, meats, and dairy products and the lower median intake of vegetables, fruits, legumes, nuts, fish, and MUFAs/SFAs ratio received a score of zero for these food items. Total MDS for each person was calculated by summing up all nine single component scores and ranged from 0 to 9. Considering religious and cultural preferences of Iranians, alcohol consumption was not taken into account in the current study for constructing the MDS. Also, considering that the Iranian society has consumed only 10 g of whole grains per day^39,40^, whole and refined grains were merged as a single group of grain in this study. In contrast to the traditional Mediterranean diet, in which whole grain is defined as a healthy food group, we classified the grain group as a non-healthy dietary group^40,41^, due to its association with chronic illnesses^40,41^.

### Assessment of outcomes

The validated Pittsburgh Sleep Quality Index (PSQI) questionnaire was utilized to evaluate the quantity and quality of sleep^42^. The validity and reliability of this tool have previously been investigated^42^. Also, the Persian version of the questionnaire has been validated among Iranian adults^43^. This questionnaire consists of 19 items which are rated on a four-point scale (0–3) and grouped into seven domains: subjective sleep quality, sleep latency, sleep duration, habitual sleep efficiency, sleep disturbances, use of sleep medications, and daytime dysfunction. Each domain is assigned a point value between 0 and 3 (0 = good and 3 = poor status). An overall score ranging from 0 to 21 was calculated using the scores of these seven domains; a higher total score indicated lower sleep quality. Participants were categorized into 3 categories: 0–3, good quality; 4–5, middle quality; and ≥ 6, poor quality^42^. In the present study, a total PSQI score of < 6 was considered as adequate sleep quality. This cutoff was based on a validation study among Iranian adults that showed a sensitivity and specificity of 93.6 and 72.2, respectively^43^. Sleep duration was obtained through the fourth question of PSQI: "How many hours do you sleep per night?" Those with less than 7 h were considered as having short sleep^44,45^. Serum BDNF levels were determined using twelve-hour fasting blood samples of participants and ELISA kits (Zellbio, Veltlinerweg, Germany). According to a previous systematic-review, BDNF range widely varies among different populations^46^, and there has not been a predefined cutoff value for categorizing BDNF levels as low and high. Prior studies have considered 10th percentile of BDNF for this classification^32,47,48^. Similar to these studies, we computed deciles of serum BDNF concentrations, and participants in the lowest decile (with serum BDNF levels < 0.47 ng/mL) were regarded to have low serum BDNF values.

### Assessment of other variables

Height and weight of participants were assessed while they stood in minimal clothing and without shoes. A wall-mounted tape-meter was used to measure height to the nearest 0.1 cm. Measurements of weight were done with the use of a body composition analyzer (Tanita MC-780MA, Tokyo, Japan). Body mass index (BMI) was calculated by dividing weight (kg) by height (m) squared. After five minutes of resting, blood pressure was measured twice in a sitting position using an accurate digital sphygmomanometer (OMRON, M3, HEM-7154-E, Japan), and the mean of the two readings was recorded for each participant. A self-reported questionnaire was used to collect information about other confounding variables such as age, sex, marital status, education, smoking habits, home ownership and other socio-economic variables, medical history of diseases, and medication or dietary supplement use. The STOP-Bang questionnaire, a self-reporting screening instrument with eight "yes or no" items about snoring, fatigue, observed apnea, high blood pressure, BMI > 35 kg/m^2^, age > 50, neck circumference > 40 cm, and male sex, was used to characterize obstructive sleep apnea (OSA). Those participants who responded positively to ≤ 2, 3–4, and ≥ 5 questions had respectively low, intermediate, and high risk of OSA. Additionally, the validated International Physical Activity Questionnaire (IPAQ) was used to assess physical activity of participants^49^.

### Statistical analysis

Kolmogorov–Smirnov test was performed to examine the normality of quantitative variables. The study participants were classified into energy-adjusted tertiles of MDS. General characteristics of the study participants across tertiles of MDS were presented as means ± SD/SE for continuous variables (age, weight, height, BMI, blood pressure, BDNF, and dietary intakes), and percentages for categorical variables (sex, physical activity levels, OSA status, socioeconomic status, smoking status, having type 2 diabetes, hypertension, and antidepressant medications usage).

To investigate the differences between MDS tertiles, one-way analysis of variance (ANOVA) and chi-square tests were performed for continuous and categorical variables. Post hoc comparisons by Bonferroni correction were performed if ANOVAs were significant. Age, sex, and energy-adjusted dietary intakes of participants across tertiles of MDS were provided using an analysis of covariance (ANCOVA).

Multivariable logistic regression was used to find a connection between tertiles of MDS and outcomes of interest. In both crude and adjusted models, the odds ratios (OR) and 95% confidence intervals (CI) for short sleeping and poor sleep quality were computed. In the first model, age, sex, and energy intake were all taken into account. In the second model, additional adjustments were made for physical activity (inactive/minimally active/ active), type 2 diabetes, hypertension, tea and coffee intake, socio-economic status, smoking status (non-smoker/former smoker/current smoker), OSA and use of antidepressant medication. BMI was added to the prior adjustments in the last model.

Crude and multivariable-adjusted models were used to obtain odds of low BDNF values (< 0.47 ng/mL) across tertiles of MDS. In the first model the effects of age, and sex were adjusted. In the second model, more adjustments were done for physical activity, type 2 diabetes, hypertension, and use of antidepressant medicine.

The lowest tertile of MDS was considered as the reference category in all models. The overall trend of odds ratios across increasing tertiles of MDS was obtained by considering these tertiles as ordinal variables in the logistic regression models. SPSS software version 26 (IBM, Chicago, IL) was used for all analyses. P-values < 0.05 (two-sided) were regarded as statistically significant.

Path analysis was also used to investigate the relationship between sleep habits as dependent variables, MD scores as an independent variable, and BDNF values as a mediating variable. Tucker Lewis index (TLI) (with a value of 0 to 1, acceptable range: values greater than 0.9), Comparative fit index (CFI) (with a value of 0 to 1, acceptable range: values greater than 0.9), and the root mean square error of approximation (RMSEA) (with a value of 0 to 1, acceptable range: values less than 0.05) were used to check the appropriateness of the model (Goodness-of-fit). R free statistical software version 3.2.2 was used for this analysis.

### Ethical approval and consent to participate

All participants provided an informed written consent. The study protocol was approved by the local Ethics Committee of Isfahan University of Medical Sciences.

### Consent to participate

Informed consent was obtained from all participants involved in the study.

## Results

This study was performed on 535 participants, with a mean age of 42.6 years and BMI of 24.9 kg/m^2^. Among participants, 54% were male. General characteristics of study participants across energy-adjusted tertiles of MDS are presented in Table 1. Participants in the top tertile of MDS were more likely to be female, older, have lower prevalence of obstructive sleep apnea (OSA) and have lower weight, and height (all P-values < 0.05). No other significant difference was observed in BMI, physical activity levels, socioeconomic status, type 2 diabetes, hypertension, systolic and diastolic blood pressure, smoking status, BDNF levels, and antidepressant medicine intake across tertiles of MDS.

**Table 1:** General characteristics of study participants across energy-adjusted tertiles of the Mediterranean diet score (n = 535).

	Tertiles of energy-adjusted MDS			P^a^
	T_1_ (n = 155) (0–3)	T_2_ (n = 219) (4–5)	T_3_ (n = 161) (6–9)	
Age (years)	39.95 ± 10.81	43.46 ± 10.97	43.91 ± 11.32	0.002
Weight (kg)	79.70 ± 15.86	75.30 ± 14.77	72.51 ± 11.80	< 0.001
Body mass index (kg/m^2^)	27.38 ± 4.54	26.84 ± 4.62	26.54 ± 3.96	0.23
Height (cm)	170.3 ± 9.02	167.34 ± 7.86	165.41 ± 8.28	< 0.001
Sex, male (%)	67.1	53.9	41.6	< 0.001
Physical activity (%)				0.28
Inactive	61.3	56.4	52.2	
Minimally active	29.7	37.6	38.4	
Active	9.0	6.0	9.4	
OSA (%)				0.04
No	58.2	66.2	74.5	
Borderline	34.9	29.5	20.3	
Yes	6.8	4.3	5.2	
Socioeconomic status levels (%)				0.67
Low	35.7	32.1	27.6	
Moderate	28.6	34.3	32.4	
High	35.7	33.6	40.0	
Smoking status (%)				0.75
Non-smoker	93.7	93.2	94.4	
Former smoker	2.1	4.2	2.8	
Current smoker	4.2	2.6	2.8	
History of type 2 diabetes, yes (%)	3.2	6.4	5.6	0.38
Hypertension, yes (%)	27.5	28.8	28.3	0.96
Antidepressant medicine, yes (%)	3.9	7.1	4.5	0.35
Systolic blood pressure (mmHg)	122.79 ± 16.91	121.60 ± 14.85	120.63 ± 16.41	0.49
Diastolic blood pressure (mmHg)	82.10 ± 9.99	83.18 ± 8.98	82.28 ± 10.65	0.52
BDNF (ng/mL)	1.17 ± 0.05	1.27 ± 0.13	1.30 ± 0.14	0.76

For continuous variables, values are mean ± SD, except for BDNF which are mean ± SE.

*BMI* body mass index, *BDNF* brain derived neurotrophic factor, *OSA* obstructive sleep apnea.

^a^Obtained from ANOVA for continuous variables and Chi-square test for categorical variables.

Dietary intakes of study participants according to the tertiles of MDS are provided in Table 2. In comparison to participants in the lowest tertile of MDS, participants in the highest tertile consumed more amounts of fruits, vegetables, legumes, nuts, fish, dietary fibers intake, omega-3 fatty acids, carbohydrates, magnesium, and less amount of meat, grains, dairy products, proteins (all P-values < 0.05). There was no significant difference in the distribution of other variables (energy, fats, tea and coffee intake, and vitamin B_1_) among tertiles of MDS.

**Table 2:** Multivariable-adjusted intakes of Mediterranean diet components and selected nutrients of study participants across energy-adjusted tertiles of the Mediterranean diet score (n = 535).

	Tertiles of energy-adjusted MDS			P^a^
	T_1_ (n = 155) (0–3)	T_2_ (n = 219) (4–5)	T_3_ (n = 161) (6–9)	
Energy (Kcal/day)	2291.32 ± 55.76	2276.35 ± 45.95	2245.63 ± 54.26	0.84
Food groups (g/day)				
Fruits	419.01 ± 24.57	539.42 ± 20.24	700.43 ± 23.91	< 0.001
Vegetables	240.11 ± 17.89	349.05 ± 14.74	436.64 ± 17.41	< 0.001
Meats	112.71 ± 4.69	90.99 ± 3.87	96.38 ± 4.57	0.002
Fish	5.35 ± 0.75	7.49 ± 0.62	10.04 ± 0.73	< 0.001
Legumes	30.87 ± 2.96	40.02 ± 2.44	45.75 ± 2.88	0.002
Nuts	7.80 ± 1.00	11.56 ± 0.83	16.20 ± 0.98	< 0.001
Grains	454.42 ± 12.91	387.85 ± 10.63	313.30 ± 12.56	< 0.001
Dairy	372.13 ± 21.26	328.38 ± 17.52	241.24 ± 20.69	< 0.001
MUFA/SFA	0.91 ± 0.02	1.02 ± 0.02	1.09 ± 0.02	< 0.001
Tea and coffee	637.68 ± 601.61	673.13 ± 50.76	784.96 ± 59.95	0.20
Other nutrients				
Proteins (% of energy)	14.93 ± 0.23	14.07 ± 0.19	13.86 ± 0.23	0.002
Fats (% of energy)	27.52 ± 0.55	26.60 ± 0.45	26.72 ± 0.54	0.41
Carbohydrates (% of energy)	58.87 ± 0.66	61.29 ± 0.55	61.98 ± 0.65	0.002
Dietary fiber (g/day)	17.08 ± 0.47	21.16 ± 0.39	25.00 ± 0.46	< 0.001
Omega-3 fatty acids (g/day)	0.34 ± 0.02	0.38 ± 0.02	0.41 ± 0.02	0.04
Vitamin B_1_ (mg/day)	2.77 ± 0.04	2.64 ± 0.03	2.53 ± 0.04	0.12
Vitamin B_3_ (mg/day)	23.65 ± 0.37	22.99 ± 0.30	22.06 ± 0.36	0.01
Magnesium (mg/day)	258.16 ± 5.17	280.70 ± 4.26	311.65 ± 5.03	< 0.001

All values are means ± standard error (SE); energy intake and macronutrients are adjusted for age and gender; all other values are adjusted for age, gender and energy intake.

*SFA* saturated fatty acids, *MUFA* monounsaturated fatty acids.

^a^Obtained from ANCOVA.

**Figure 1:**
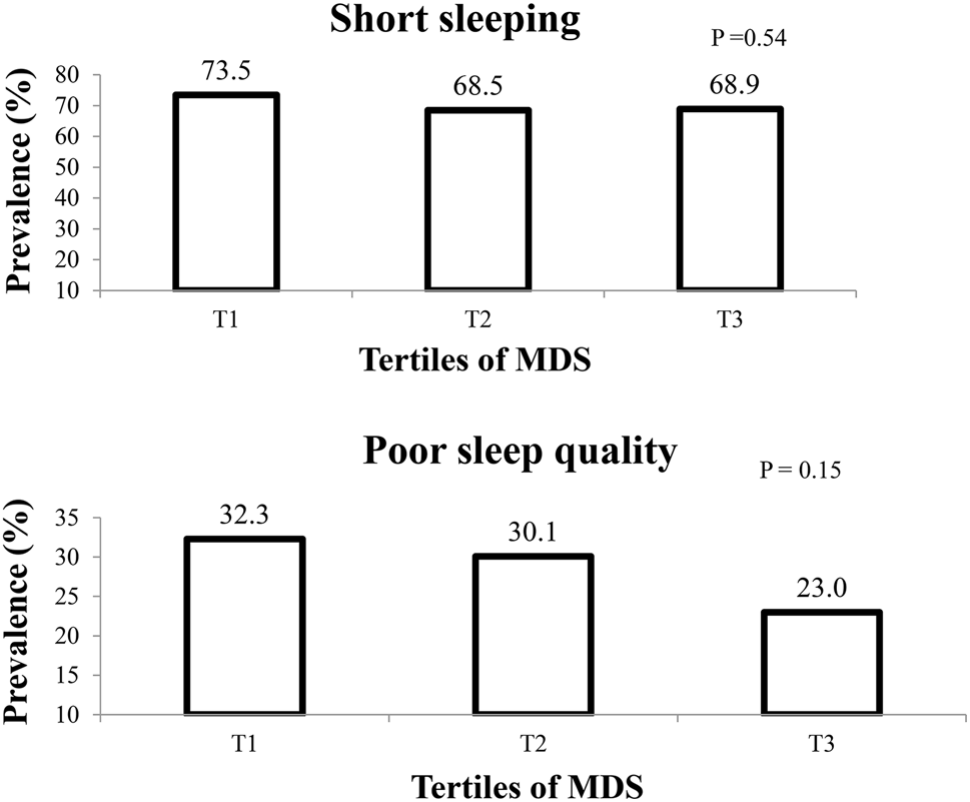
Prevalence of short sleeping and poor sleep quality in energy-adjusted tertiles of MDS.

Among study participants, 70.1% had short sleep and 28.6% had poor sleep quality. The prevalence of individuals with short sleep and poor sleep quality across tertiles of MDS is shown in Fig. 1. In the lowest, middle, and highest tertile of MDS, 73.5%, 68.5%, and 68.9% of individuals had short sleep duration (< 7 h), (P = 0.54). Also, no difference in prevalence of poor sleep quality was observed across tertiles of MDS (32.3%, 30.1%, and 23.0%, P = 0.15).

Crude and multivariable-adjusted ORs for short sleeping and poor sleep quality across tertiles of MDS are provided in Table 3. The highest adherence to MDS was not significantly related to odds of having short sleep duration (< 7 h/night) in the crude model (OR = 0.80, 95% CI: 0.49–1.30). Following the adjustment of potential confounders, participants in the top tertile of MDS had lower odds of having short sleep than those in the bottom tertile (OR = 0.44, 95% CI: 0.21–0.91). A significant decreasing trend (P_trend_ < 0.03) was also found for the odds of having short sleep across MDS tertiles. Poor sleep quality and MDS did not have a significant relation in the crude model (OR = 0.57, 95% CI: 0.30–1.10). However, after adjustment for potential confounder, individuals in the top tertile of MDS had lower odds of having poor sleep quality (OR = 0.46, 95% CI: 0.22–0.96). The relations between each component of MDS and sleep quality and duration are also presented in Supplementary Tables 1 and 2. Higher legumes intakes were marginally associated with lower odds of having short sleep duration, while no other significant relation was found. Legumes intake was also inversely related to odds of having poor-quality of sleep.

**Table 3:** Multivariable-adjusted odds ratio for short sleeping and poor sleep quality across energy-adjusted tertiles of the Mediterranean dietary score (n = 535).

	Tertiles of energy-adjusted MDS			P_trend_^a^
	T_1_ (0–3)	T_2_ (4–5)	T_3_ (6–9)	
Short sleeping				
Participants/cases (n)	155/114	219/150	161/111	
Crude	1.00	0.78 (0.50–1.24)	0.80 (0.49–1.30)	0.38
Model 1	1.00	0.77 (0.48–1.22)	0.81 (0.49–1.34)	0.43
Model 2	1.00	0.50 (0.25–0.99)	0.43 (0.21–0.90)	0.03
Model 3	1.00	0.51 (0.26–1.00)	0.44 (0.21–0.91)	0.03
Poor sleep quality				
Participants/cases (n)	155/50	219/66	161/37	
Crude	1.00	0.76 (0.42–1.39)	0.57 (0.30–1.10)	0.09
Model 1	1.00	0.73 (0.40–1.34)	0.53 (0.27–1.05)	0.07
Model 2	1.00	0.81 (0.42–1.55)	0.46 (0.22–0.96)	0.04
Model 3	1.00	0.81 (0.42–1.55)	0.46 (0.22–0.96)	0.04

All values are odds ratios and 95% confidence intervals. P_trend_ was obtained by the use of tertiles of MDS as an ordinal variable in the model. Model 1: Adjusted for age, gender, energy intake. Model 2: More adjustments for physical activity levels, socioeconomic status, type 2 diabetes, hypertension, tea and coffee intake, use of antidepressant medicine, OSA, and smoking status. Model 3: Further adjustment for BMI.

^a^P trend was obtained by considering the tertiles of MDS as ordinal variable.

Multivariable-adjusted ORs for each domain of sleep quality across tertiles of MDS are provided in Table 4. Regarding the particular indicators of sleep quality, individuals in the highest tertile of MDS compared to the lowest tertile had lower odds for self-reported poor sleep quality (OR = 0.32, 95% CI: 0.10–0.96), sleep latency (OR = 0.29, 95% CI: 0.10–0.82) and daytime dysfunction (OR = 0.37, 95% CI: 0.14–0.99), after adjustment for all potential confounders.

**Table 4:** Multivariable-adjusted odds ratio for individual domains of sleep quality across energy-adjusted tertiles of the Mediterranean dietary score n = 535).

	Tertiles of energy-adjusted MDS			P_trend_^a^
	T_1_ (n = 155) (0–3)	T_2_ (n = 219) (4–5)	T_3_ (n = 161) (6–9)	
Subjective sleep quality				
Cases (n)	23	21	14	
Crude	1.00	0.46 (0.20–1.06)	0.38 (0.15–0.97)	0.03
Model 1	1.00	0.44 (0.18–1.07)	0.41 (0.15–1.12)	0.06
Model 2	1.00	0.51 (0.20–1.32)	0.32 (0.10–0.96)	0.03
Model 3	1.00	0.52 (0.20–1.33)	0.32 (0.10–0.96)	0.04
Sleep latency				
Cases (n)	27	26	18	
Crude	1.00	0.72 (0.33–1.58)	0.54 (0.22–1.31)	0.17
Model 1	1.00	0.63 (0.28–1.41)	0.43 (0.17–1.10)	0.07
Model 2	1.00	0.60 (0.25–1.48)	0.30 (0.11–0.84)	0.02
Model 3	1.00	0.61 (0.25–1.49)	0.29 (0.10–0.82)	0.02
Sleep duration				
Cases (n)	69	80	64	
Crude	1.00	0.50 (0.28–0.88)	0.64 (0.35–1.16)	0.14
Model 1	1.00	0.46 (0.26–0.84)	0.60 (0.32–1.12)	0.11
Model 2	1.00	0.43 (0.27–0.80)	0.54 (0.28–1.05)	0.07
Model 3	1.00	0.45 (0.24–0.84)	0.55 (0.28–1.08)	0.08
Habitual sleep efficiency^b^				
Cases (n)	0	1	1	
Crude	1.00	–	–	–
Model 1	1.00	–	–	–
Model 2	1.00	–	–	–
Model 3	1.00	–	–	–
Sleep disturbances				
Cases (n)	16	27	13	
Crude	1.00	1.01 (0.38–2.68)	0.98 (0.35–2.72)	0.96
Model 1	1.00	0.87 (0.32–2.37)	0.73 (0.25–2.16)	0.57
Model 2	1.00	0.91 (0.31–2.66)	0.70 (0.22–2.29)	0.56
Model 3	1.00	0.91 (0.31–2.66)	0.70 (0.22–2.29)	0.56
Use of sleep medications				
Cases (n)	5	9	4	
Crude	1.00	0.60 (0.13–2.73)	0.72 (0.16–3.33)	0.66
Model 1	1.00	0.42 (0.09–2.05)	0.37 (0.07–1.93)	0.24
Model 2	1.00	0.42 (0.08–2.38)	0.41 (0.06–2.79)	0.35
Model 3	1.00	0.41 (0.07–2.35)	0.40 (0.06–2.74)	0.34
Daytime dysfunction				
Cases (n)	27	29	17	
Crude	1.00	0.67 (0.32–1.41)	0.43 (0.18–1.02)	0.05
Model 1	1.00	0.66 (0.30–1.43)	0.44 (0.18–1.10)	0.07
Model 2	1.00	0.64 (0.28–1.43)	0.37 (0.14–0.99)	0.05
Model 3	1.00	0.64 (0.28–1.44)	0.37 (0.14–0.99)	0.05

All values are odds ratios and 95% confidence intervals. P_trend_ was obtained by the use of tertiles of MDS as an ordinal variable in the model. For each domain of sleep quality the odds was reported for score 2–3 of that domain (as poor quality); while score 0–1 was considered as the reference category. Model 1: adjusted for age, gender, energy intake. Model 2: more adjustments for physical activity levels, socioeconomic status, type 2 diabetes, hypertension, tea and coffee intake, use of antidepressant medicine, OSA, and smoking status. Model 3: further adjustment for BMI.

^a^P trend was obtained by considering the tertiles of MDS as ordinal variable.

^b^OR (95% CI) could not be calculated, due to not having cases in one tertile.

Multivariable-adjusted ORs for short sleeping and poor sleep quality across tertiles of MDS, stratified by BMI categories are shown in Table 5. Participants in the highest tertile of MDS compared to the lowest one had lower odds of having short sleep duration among individuals with overweight or obesity (OR = 0.36, 95% CI: 0.14–0.94), but not in those with normal weight (OR = 0.26, 95% CI: 0.06–1.15). Also, there was a statistically significant descending trend for this relation across tertiles of MDS (P_trend_ = 0.04). With regard to sleep quality, participants with normal weight in the top tertile of MDS had lower odds for having poor sleep quality (OR = 0.16, 95% CI: 0.03–0.84); however, there was no significant relationship between sleep quality and MDS among individuals with overweight/obesity (OR = 0.58, 95% CI: 0.23–1.45).

**Table 5:** Multivariable-adjusted odds ratio for short sleeping and poor sleep quality across energy-adjusted tertiles of the Mediterranean dietary score, stratified by BMI categories (n = 535).

	Tertiles of energy-adjusted MDS			P_trend_^a^
	T_1_ (n = 155) (0–3)	T_2_ (n = 219) (4–5)	T_3_ (n = 161) (6–9)	
Short sleeping				
Normal weight (BMI < 25)				
Crude	1.00	0.76 (0.34–1.70)	0.73 (0.30–1.73)	0.47
Model 1	1.00	0.70 (0.30–1.60)	0.71 (0.29–1.74)	0.46
Model 2	1.00	1.28 (0.34–4.85)	0.26 (0.06–1.15)	0.09
Overweight and obese (BMI ≥ 25)				
Crude	1.00	0.79 (0.45–1.38)	0.83 (0.46–1.51)	0.56
Model 1	1.00	0.80 (0.45–1.41)	0.86 (0.46–1.60)	0.66
Model 2	1.00	0.40 (0.17–0.96)	0.36 (0.14–0.94)	0.04
Poor sleep quality				
Normal weight (BMI < 25)				
Crude	1.00	0.79 (0.29–2.14)	0.24 (0.06–1.02)	0.06
Model 1	1.00	0.73 (0.26–2.03)	0.22 (0.05–0.96)	0.05
Model 2	1.00	0.54 (0.16–1.79)	0.16 (0.03–0.84)	0.03
Overweight and obese (BMI ≥ 25)				
Crude	1.00	0.80 (0.38–1.71)	0.78 (0.36–1.67)	0.52
Model 1	1.00	0.81 (0.37–1.78)	0.76 (0.33–1.75)	0.53
Model 2	1.00	0.91 (0.40–2.10)	0.58 (0.23–1.45)	0.24

All values are odds ratios and 95% confidence intervals. P_trend_ was obtained by the use of tertiles of MDS as an ordinal variable in the model. Model 1: adjusted for age, gender, energy intake. Model 2: more adjustments for physical activity levels, socioeconomic status, type 2 diabetes, hypertension, tea and coffee intake, and use of antidepressant medicine, OSA, smoking status.

^a^P trend was obtained by considering the tertiles of MDS as ordinal variable.

**Figure 2:**
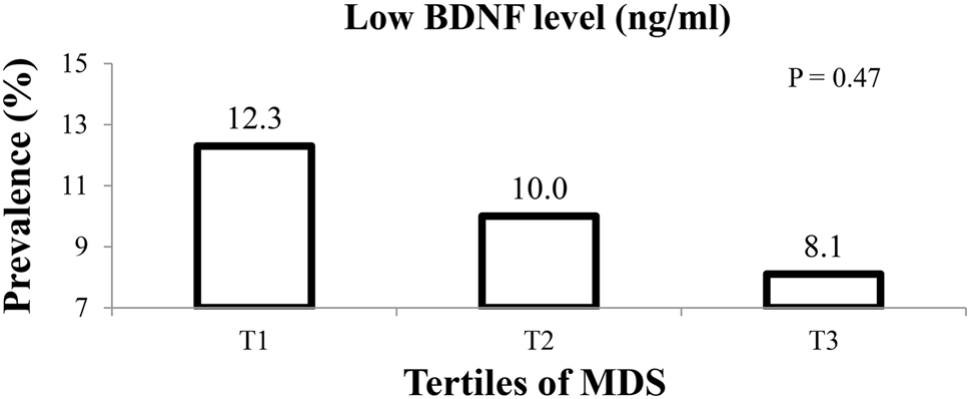
Prevalence of low serum BDNF values across energy-adjusted tertiles of the MDS.

The mean serum BDNF among the study population was 1.25 (± 0.07 SE) ng/mL. The prevalence of low BDNF level across energy-adjusted tertiles of MDS is presented in Fig. 2. No differences were observed in prevalence of having low serum BDNF (< 0.47 ng/mL or lowest decile) between the highest and the lowest tertile of MDS (8.1% vs. 12.3%, P = 0.47).

Table 6 presents multivariate-adjusted odds ratio (ORs) and 95% confidence intervals (CIs) for the prevalence of low BDNF values across MDS tertiles. The association between MDS and the odds of low BDNF level was insignificant in the crude model (OR = 0.65, 95% CI: 0.31–1.37). After controlling for potential confounders, this association was still insignificant (OR = 0.57, 95% CI: 0.26–1.25).

**Table 6:** Multivariate adjusted odds ratio (OR) and 95% confidence interval (CI) for low BDNF level (< 0.47 ng/mL, 1st decile) across tertiles of the Mediterranean dietary score (n = 535).

	Tertiles of energy-adjusted MDS			P_trend_^a^
	T_1_ (n = 155) (0–3)	T_2_ (n = 219) (4–5)	T_3_ (n = 161) (6–9)	
Cases (n)	19	22	13	
Crude	1.00	0.75 (0.38–1.45)	0.65 (0.31–1.37)	0.25
Model 1	1.00	0.71 (0.36–1.41)	0.59 (0.27–1.28)	0.18
Model 2	1.00	0.70 (0.35–1.40)	0.57 (0.26–1.25)	0.17

All values are odds ratios and 95% confidence intervals. P_trend_ was obtained by the use of tertiles of MDS as an ordinal variable in the model. Model 1: adjusted for age, gender. Model 2: more adjustments for physical activity levels, type 2 diabetes, hypertension, and use of antidepressant medicine.

^a^P trend was obtained by considering the tertiles of MDS as ordinal variable.

The prevalence of low BDNF values was not significantly different between individuals with short and normal sleep duration (11.0% vs. 9.8%, P = 0.39). Furthermore, the prevalence of low level of BDNF was not significantly different between participants with poor sleep quality and those with good or moderate quality of sleep (12.4% vs. 9.2%, P = 0.16) (data not shown).

As shown in Supplementary Fig. 1, path analysis revealed a significant positive association between MD scores and BDNF values. However, BDNF values were not significantly related to sleep quality or quantity (as continuous outcome variables), indicating no significant indirect relation. The model had appropriate goodness-of-fit (TLI = 1, CFI = 1, RMSEA = 0).

## Discussion

In the current cross-sectional study, Iranian individuals with higher adherence to the MDS were less likely to have short sleep duration and poor sleep quality. Considering individual domains of sleep quality, MDS was inversely related to subjective sleep quality, sleep latency, and daytime dysfunction. Also, stratified analysis based on BMI categories indicated that adherence to MDS was inversely related to having short sleep duration in individuals with overweight/obesity. However, this inverse association was significant in individuals who had normal weight when poor sleep quality was considered as the outcome. As far as we know, the present study was the first population-based study addressing the association between the Mediterranean diet pattern, sleep habits, and brain-derived neurotrophic factor (BDNF) among Iranian adults.

Inadequate levels of sleep duration and quality are associated with an increased risk of cardio-metabolic disorders such as diabetes, and hypertension, and mortality^50–52^. Furthermore, sleep problems have been suggested to negatively affect quality of life^53^. We observed that individuals with higher adherence to MD might have better sleep duration and quality. Therefore, clinicians could recommend individuals to shift their dietary intakes to a Mediterranean-style diet in order to improve their sleep health.

Our current study revealed that adults who have the greatest adherence to the MD might have decreased odds of having short sleep duration and low sleep quality. Findings from similar studies are contradictory. Similar to our findings, higher adherence to MD was associated with better sleep quality in a cross-sectional study on Italian adults^54^. Additionally, a prospective cohort study on American women found that greater adherence to MD could be related to better overall sleep quality at 1-y follow-up^55^. A cross-sectional study on Spanish pregnant women showed similar results^56^. Another cross-sectional study on Costa Rican adults showed that women with lower adherence to MD were more likely to have shorter sleep duration^57^. Conversely, a cross-sectional study among Swedish elder men did not find a significant relation between MD and sleep quality^58^. Discrepancies among available studies might originate in different questionnaires and methods that were used to assess MD adherence and sleep health. Also, different study designs and participants, as well as different approach for considering confounders could be other reasons for observed controversies.

In the current study, greater adherence to the Mediterranean diet, in participants with overweight/obesity was associated with decreased odds of having short sleep duration. When we considered poor sleep quality as the outcome, this association was significant among adults with normal weight, but not those with overweight/obesity. In line with our finding, a cohort study among Italian individuals found a relation between MD and sleep quality only among participants with normal- or overweight^11^. A possible reason is that individuals with obesity might have other unhealthy lifestyle habits that neutralize the beneficial effect of MD; and we were unable to take all of these confounders into consideration in our analysis. Although prior evidence has shown a bidirectional association between obesity and sleep quality^59^, the relation between MD and sleep quality and quantity has not been adequately compared among individuals with different weight status. Therefore, future prospective studies are required explaining to what extend the relation between MD and sleep quality/quantity might differ among individuals with different BMI categories.

Earlier studies have suggested lower BDNF concentrations in patients with neurodegenerative diseases such as Alzheimer^60^, and Parkinson^61^. Also, a meta-analysis showed that individuals who had depression might have decreased levels of BDNF^62^. The relationship between BDNF and some metabolic disorders such as obesity and metabolic syndrome have additionally been addressed^63,64^. But, the association between BDNF and sleep quality/quantity has not been widely investigated. A recent discovering aspect of BDNF is its causal role in sleep regulation that makes this molecule as a regulator of stress in central nervous system (CNS) and neural plasticity. Lower BDNF levels have been reported in those who had short sleep duration compared to those who did not^27^. We did not find significant associations between BDNF and sleep quality or quantity. It is worth noting that our sample size was not calculated based on such hypothesis as we had financial limitations. Also, several factors might affect this association that we were not able to take them into account as confounders. These issues might explain our null results. Therefore, larger studies along with considering various underlying factors are required in this field.

The association between usual or long-term dietary intakes and BDNF levels was less studied, and most of the studies in this field were interventional. Given the inverse relation between MD and poor sleep quality/quantity^55–57^ and the possible role of BDNF, we hypothesized that individuals who had higher adherence to MD might be less likely to have low BDNF values. We failed to find any association between MD and low-BDNF or between low-BDNF and sleep quality/quantity. In contrast, a previous trial on Spanish adults showed positive effects of MD on BDNF values^32^. A possible explanation is the differences in the study design and populations. In a low-income country, such as Iran, adherence to MD is lower than a European country. Therefore, a high adherence to MD in Iran might be categorized as a low or middle adherence in a western country.

We also did pathway analysis to examine the possible mediating role of BDNF in the relation of MD and sleep quality/quantity. A positive association was found between MD scores and BDNF levels. However, BDNF was not associated with sleep quality or quantity. This suggests that BDNF would not be a mediating factor in the relation between MDS and sleep quality/quantity. However, the sample size and differences in statistical approaches should be acknowledged. More studies are warranted to examine this mediating effect.

MD might exert its effect on sleep duration and quality through several possible mechanisms. This diet contains seeds, nuts, fish, and poultry that are rich in tryptophan, an amino acid that regulates circadian rhythms and also known as an effective sleep promoter^65,66^. Considering a high content of antioxidant components and anti-inflammatory compounds, the MD pattern has neuroprotective effects and can decrease oxidative stress^10^. Inflammation and oxidative stress, which might occur in the body, have been suggested as two major causes of poor sleep quality^67,68^. MD contain sleep-promoting foods which could improve hormones such as serotonin and melatonin; and it has been recognized that melatonin and serotonin are connected to sleep–wake brain centers^15,65^ and low serotonin levels can lead to poor sleep quality/ quantity^69^. On the other hand, decreased BDNF level could result in decreased serotonin signaling and consequently poor sleep quality^62^. In addition, fruits and vegetables are rich sources of fiber and other essential nutrients that can have beneficial impacts on the microbial composition of the gut^70^, which could in turn help improving sleep quality^71^. Moreover, higher adherence to MD could be related to lower inflammatory cytokines including C-reactive protein (CRP), interleukin-1 (IL-1), tumor necrosis factor-α, and IL-6^72,73^. Increased levels of these inflammatory markers have been linked to poor sleep quality among different groups of people^65,73^.

Some strengths of this study should be highlighted. This is one of the first studies among a Middle Eastern society that examined MD in relation to sleep quality and duration, as well as serum BDNF levels. The study sample was selected by use of a multistage cluster random sampling method; so, our population could be representative of the Iranian adult population, and the findings could be generalizable to general adult population. We also used valid questionnaires to assess dietary intakes and sleep health status of participants. However, several limitations should be taken into account, while interpreting our findings. The main limitation of our study was its cross-sectional design, which made it difficult to infer a causal connection between MDS and sleep outcomes. Thus, prospective cohort studies are warranted to confirm a causal relation between MDS and sleep outcomes. Despite evaluating dietary intakes by use of a validated FFQ, recall bias or other possible reporting biases were inevitable and might have affected our findings. Although we considered some possible confounders in our analyses, residual confounding cannot be ruled out and might have affected the results. COVID-19 pandemic resulted in impaired sleep quality as suggested by previous studies^74^, that could consequently affect our results. Also, studies have shown that BDNF levels might be negatively related to COVID^75,76^. However, this might not be considerable as the data were collected when the pandemic condition was stable and quarantine was not mandatory. Finally, although our study population was a representative sample of Iranians, we cannot extrapolate our findings to other nations. Therefore, more studies in different populations around the world are warranted. Also, future studies should focus on how other risk factors such as age, gender or BMI could affect the relation between MD and sleep disturbances.

In conclusion, this cross-sectional study indicated that adults with higher adherence to the Mediterranean diet had significantly lower odds of having short sleep duration and poor sleep quality. This association was significant in individuals with overweight or obesity for sleep duration and those with normal weight for sleep quality. We found that BDNF would not be an intermediate molecule for this connection. Prospective studies are required to affirm these results.

### Supplementary Information

Supplementary Information.

## Data Availability

The data that support the findings of this study are available from the corresponding author upon reasonable request.

## Abbreviations

FFQ: Food Frequency Questionnaire
OR: Odds ratios
95% CI: 95% Confidence interval
BMI: Body mass index
MUFA: Mono unsaturated fatty acids
PUFA: Poly unsaturated fatty acids
SFA: Saturated fatty acids
ANOVA: Analysis of variance
ANCOVA: Analysis of covariance
SPSS: Statistical package for the social sciences
SD: Standard deviation
SE: Standard error
IPAQ: International Physical Activity Questionnaire
MDS: Mediterranean Diet Score
MD: Mediterranean diet
BDNF: Brain-derived neurotrophic factor
PSQI: Pittsburgh Sleep Quality Index
OSA: Obstructive sleep apnea
STOP-Bang: Snoring, Tiredness, Observed apnea, blood Pressure, Body mass index, Age, Neck circumference and Gender
