# Supplementary material for: Association between Mediterranean dietary pattern with sleep duration, sleep quality and brain derived neurotrophic factor (BDNF) in Iranian adults
Source: Sci Rep. 2023 Aug 18;13:13493. doi: 10.1038/s41598-023-40625-4 (PMC10439234; doi:10.1038/s41598-023-40625-4)
Supplement: Supplementary file 1 — Supplementary Information. [file 41598_2023_40625_MOESM1_ESM.docx]

**Supplementary Table 1. Multivariable-adjusted odds ratio for having short duration of sleep across tertiles of MDS components ^1^**

|  | Tertiles of energy-adjusted MDS components | | |  |
| --- | --- | --- | --- | --- |
|  | T_1_ | T_2_ | T_3_ | P_trend_ |
| **Dairy** |  |  |  |  |
| Crude | 1.00 | 1.10 (0.69-1.74) | 0.83 (0.53-1.30) | 0.42 |
| Multivariable-adjusted^2^ | 1.00 | 1.25 (0.62-2.50) | 1.19 (0.61-2.32) | 0.65 |
| **Fish** |  |  |  |  |
| Crude | 1.00 | 1.06 (0.68-1.67) | 1.08 (0.69-1.71) | 0.73 |
| Multivariable-adjusted^2^ | 1.00 | 0.79 (0.39-1.63) | 0.81 (0.41-1.60) | 0.58 |
| **Fruits** |  |  |  |  |
| Crude | 1.00 | 1.29 (0.81-2.04) | 0.97 (0.62-1.52) | 0.91 |
| Multivariable-adjusted^2^ | 1.00 | 1.91 (0.98-3.73) | 1.17 (0.59-2.35) | 0.56 |
| **Vegetables** |  |  |  |  |
| Crude | 1.00 | 0.83 (0.53-1.32) | 0.78 (0.50-1.24) | 0.30 |
| Multivariable-adjusted^2^ | 1.00 | 1.23 (0.61-2.46) | 0.68 (0.35-1.34) | 0.25 |
| **Legumes** |  |  |  |  |
| Crude | 1.00 | 0.76 (0.48-1.21) | 0.67 (0.42-1.05) | 0.08 |
| Multivariable-adjusted^2^ | 1.00 | 0.84 (0.41-1.74) | 0.52 (0.26-1.03) | 0.05 |
| **Nuts** |  |  |  |  |
| Crude | 1.00 | 0.96 (0.61-1.50) | 1.12 (0.71-1.76) | 0.64 |
| Multivariable-adjusted^2^ | 1.00 | 0.93 (0.47-1.86) | 0.67 (0.34-1.32) | 0.24 |
| **Meats** |  |  |  |  |
| Crude | 1.00 | 0.89 (0.57-1.38) | 1.21 (0.77-1.93) | 0.42 |
| Multivariable-adjusted^2^ | 1.00 | 0.75 (0.38-1.46) | 1.24 (0.62-2.50) | 0.53 |
| **Grains** |  |  |  |  |
| Crude | 1.00 | 1.21 (0.77-1.89) | 1.42 (0.90-2.24) | 0.13 |
| Multivariable-adjusted^2^ | 1.00 | 0.84 (0.44-1.62) | 1.06 (0.52-2.15) | 0.91 |
| **MUFA:SFA ratio** |  |  |  |  |
| Crude | 1.00 | 0.84 (0.54-1.32) | 1.09 (0.69-1.72) | 0.73 |
| Multivariable-adjusted^2^ | 1.00 | 1.08 (0.57-2.05) | 1.21 (0.61-2.42) | 0.58 |

^1^All values are odds ratios and 95% confidence intervals.

^2^Adjusted for age, gender, energy intake, physical activity levels, socioeconomic status, type 2 diabetes, hypertension, tea and coffee intake, use of antidepressant medicine, OSA, smoking status and BMI.

**Supplementary Table 2. Multivariable-adjusted odds ratio for having poor-quality of sleep across tertiles of MDS components ^1^**

|  | Tertiles of energy-adjusted MDS components | | |  |
| --- | --- | --- | --- | --- |
|  | T_1_ | T_2_ | T_3_ | P_trend_ |
| **Dairy** |  |  |  |  |
| Crude | 1.00 | 1.27 (0.67-2.40) | 1.07 (0.57-2.00) | 0.86 |
| Multivariable-adjusted^2^ | 1.00 | 1.40 (0.70-2.83) | 1.26 (0.64-2.49) | 0.53 |
| **Fish** |  |  |  |  |
| Crude | 1.00 | 1.02 (0.53-1.96) | 1.03 (0.55-1.92) | 0.93 |
| Multivariable-adjusted^2^ | 1.00 | 0.99 (0.49-2.03) | 0.95 (0.48-1.88) | 0.87 |
| **Fruits** |  |  |  |  |
| Crude | 1.00 | 1.17 (0.64-2.16) | 0.88 (0.47-1.67) | 0.71 |
| Multivariable-adjusted^2^ | 1.00 | 1.21 (0.63-2.33) | 0.85 (0.41-1.75) | 0.70 |
| **Vegetables** |  |  |  |  |
| Crude | 1.00 | 0.81 (0.44-1.50) | 0.74 (0.40-1.37) | 0.33 |
| Multivariable-adjusted^2^ | 1.00 | 0.74 (0.38-1.45) | 0.67 (0.33-1.36) | 0.27 |
| **Legumes** |  |  |  |  |
| Crude | 1.00 | 0.63 (0.35-1.14) | 0.23 (0.12-0.47) | <0.001 |
| Multivariable-adjusted^2^ | 1.00 | 0.49 (0.25-0.99) | 0.19 (0.09-0.41) | <0.001 |
| **Nuts** |  |  |  |  |
| Crude | 1.00 | 1.21 (0.66-2.23) | 0.81 (0.43-1.54) | 0.54 |
| Multivariable-adjusted^2^ | 1.00 | 1.23 (0.62-2.44) | 0.80 (0.40-1.61) | 0.51 |
| **Meats** |  |  |  |  |
| Crude | 1.00 | 1.01 (0.54-1.88) | 1.00 (0.53-1.88) | 0.99 |
| Multivariable-adjusted^2^ | 1.00 | 0.90 (0.46-1.79) | 1.06 (0.53-2.09) | 0.87 |
| **Grains** |  |  |  |  |
| Crude | 1.00 | 1.50 (0.80-2.78) | 1.23 (0.65-2.36) | 0.52 |
| Multivariable-adjusted^2^ | 1.00 | 1.60 (0.81-3.13) | 1.28 (0.62-2.66) | 0.50 |
| **MUFA:SFA ratio** |  |  |  |  |
| Crude | 1.00 | 1.01 (0.54-1.88) | 1.49 (0.79-2.82) | 0.22 |
| Multivariable-adjusted^2^ | 1.00 | 0.88 (0.45-1.72) | 1.20 (0.61-2.35) | 0.59 |

^1^All values are odds ratios and 95% confidence intervals.

^2^Adjusted for age, gender, energy intake, physical activity levels, socioeconomic status, type 2 diabetes, hypertension, tea and coffee intake, use of antidepressant medicine, OSA, smoking status and BMI.

**Supplementary Figure 1.** Path analysis considering MED diet score as the exposure, sleep quality and duration as the outcomes and BDNF values as a mediating factor. (** indicates P-value<0.001 and * indicates P-value<0.05)
